# Genomic Characterization of Antimicrobial Resistance, Virulence, and Phylogeny of the Genus *Ochrobactrum*

**DOI:** 10.3390/antibiotics9040177

**Published:** 2020-04-13

**Authors:** Yael Yagel, Stephanie Sestito, Yair Motro, Anat Shnaiderman-Torban, Boris Khalfin, Orly Sagi, Shiri Navon-Venezia, Amir Steinman, Jacob Moran-Gilad

**Affiliations:** 1 Microbiology, Advanced Genomics and Infection Control Applications Laboratory (MAGICAL), Department of Health Systems Management, School of Public Health, Faculty of Health Sciences, Ben-Gurion University of the Negev, Beer-Sheva 8410501, Israel; ygrushka@gmail.com (Y.Y.); sestito@post.bgu.ac.il (S.S.); motroy@post.bgu.ac.il (Y.M.); boriskh83@gmail.com (B.K.); 2 Koret School of Veterinary Medicine, The Robert H. Smith Faculty of Agriculture, Food and Environment, The Hebrew University of Jerusalem, Rehovot 761001, Israel; ashnaiderman@gmail.com (A.S.-T.); amirst@savion.huji.ac.il (A.S.); 3 Microbiology Lab, Soroka University Medical Center, Beer-Sheva 8410101, Israel; OrliSa@clalit.org.il; 4 Department of Molecular Biology and the Adelson School of Medicine, Ariel University, Ariel 40700, Israel; shirinv@ariel.ac.il

**Keywords:** β-lactamase, whole-genome sequencing, antimicrobial resistance, veterinary, phylogeny

## Abstract

*Ochrobactrum* is a ubiquitous Gram-negative microorganism, mostly found in the environment, which can cause opportunistic infections in humans. It is almost uniformly resistant to penicillins and cephalosporins through an AmpC-like β-lactamase enzyme class (OCH). We studied 130 assembled genomes, of which 5 were animal-derived isolates recovered in Israel, and 125 publicly available genomes. Our analysis focused on antimicrobial resistance (AMR) genes, virulence genes, and whole-genome phylogeny. We found that 76% of *Ochrobactrum* genomes harbored a *bla*_OCH_ β-lactamase gene variant, while 7% harbored another AmpC-like gene. No virulence genes other than lipopolysaccharide-associated genes were found. Core genome multilocus sequence typing clustered most samples to known species, but neither geographical clustering nor isolation source clustering were evident. When analyzing the distribution of different *bla*_OCH_ variants as well as of the *bla*_OCH_-deficient samples, a clear phylogenomic clustering was apparent for specific species. The current analysis of the largest collection to date of *Ochrobactrum* genomes sheds light on the resistome, virulome, phylogeny, and species classification of this increasingly reported human pathogen. Our findings also suggest that *Ochrobactrum* deserves further characterization to underpin its evolution, taxonomy, and antimicrobial resistance.

## 1. Introduction

The genus *Ochrobactrum* comprises a group of non-fermenting, aerobic, Gram-negative bacilli that are environmentally ubiquitous [1]. These bacteria have been isolated from soil, water, animal, and human sources—especially in gastric biopsies and from dialysis fluid [2,3,4,5]. While these species are infrequently the cause of human disease, they are associated with opportunistic central catheter-associated infections in immunocompromised human hosts [6]. Other reports of infections in humans include endocarditis, peritonitis, meningitis, osteomyelitis, endophthalmitis, septic arthritis, and bacteremia [7,8,9,10,11,12,13]. Additionally, case reports indicate this pathogen has the ability to affect even immunocompetent human hosts, albeit with limited pathogenicity [14,15,16]. These features, in combination with the genus’ close phylogenetic proximity to *Brucella* spp., a highly pathogenic group, have recently drawn considerable attention to the *Ochrobactrum* genus.

The antimicrobial resistance of this genus is of particular interest. Resistance to most cephalosporins and penicillins is known to be widespread throughout *Ochrobactrum* spp., and all species within this genus exhibit an ‘AmpC phenotype’ of β-lactam resistance (i.e., resistance to cephalosporins, cephamycins, and β-lactamase inhibitors) [17,18,19]. One report found a group of six *Ochrobactrum anthropi* isolates which were also carbapenem-resistant [20]. Susceptibility to colistin appears to be species-related, as *O. anthropi* is usually susceptible to polymyxins, whereas *O Ochrobactrum intermedium* is usually, though not always, resistant to this class of drugs [3,18,21]. These findings should be interpreted with caution in the absence of a globally standardized and validated method for determining colistin resistance in this microorganism. The genomic basis for β-lactam resistance of *Ochrobactrum* was found to be a chromosomal gene resembling an Ambler class C β-lactamase gene, encoding an AmpC-like enzyme that was named OCH-1 and its variants OCH-2 through OCH-7 [22,23]. Interestingly, later studies found the presence of OCH genes in 83% of tested isolates, even though all strains expressed an AmpC-resistance phenotype [24]. 

Despite the increasing interest in *Ochrobactrum*, both identification at the genus level and species differentiation are still challenging. Available commercial systems such as matrix-assisted laser desorption/ionization time-of-flight mass spectrometry (MALDI-TOF MS) have limited reference databases, and several reports of misidentification of *Brucella* as *Ochrobactrum* by MALDI-TOF [25,26], as well as VITEK 2 and 16S *rRNA* gene sequencing, have been published [27,28]. Using the 16S *rRNA* gene for species identification has been shown to have limited discriminatory ability [21], leading some authors to propose the use of a combination of several sequenced genes such as 16S *rRNA* and *recA* as the standard for species assignment [29]. Moreover, *recA* sequencing [29] and multilocus sequence typing (MLST) [30] were previously used to study the inter- and intra-species phylogenetic relations. Nevertheless, there is currently no accepted scheme for phylogenetic typing of this genus, despite the increasing availability of whole-genome sequencing (WGS)-based tools such as core genome MLST (cgMLST).

Several studies have used WGS for the exploration of environmentally important metabolic pathways in *Ochrobactrum* isolates [31,32,33]. Additionally, WGS recently allowed the reclassification of *Ochrobactrum lupini* as a heterotypic synonym of *O. anthropi* [34], showing promise for future application of WGS as an accurate and powerful tool for species discrimination and taxonomical assignment. Given the taxonomical uncertainties, the lack of a widely accepted phylogenetic typing scheme, in combination with this group’s yet to be explored antimicrobial resistance (AMR), we sought to utilize WGS analysis to better characterize the resistance and virulence determinants (resistome and virulome, respectively), as well as the phylogeny of this potential human pathogen using the largest genomic dataset of *Ochrobactrum* studied to date.

## 2. Results

A total of 130 whole-genome sequences of *Ochrobactrum* isolates were available for analysis. This dataset comprises five novel isolates obtained from veterinary surveillance cultures in animals in Israel, 25 raw sequences obtained and assembled from the Sequence Read Archive (SRA) database, and 100 ready assemblies downloaded from the PATRIC database. Only assemblies of sufficient quality were included. Table 1 contains relevant details of our five new genomes as well as information on the sample source. Of the 125 publicly available genomes, 76% had metadata regarding the source of isolation, and 67% had data on geographic location (Table S1), but additional data on phenotypic antimicrobial resistance were not available. Of all samples with known isolation sources, 51% were recovered from the environment, and 22% were isolated from human samples. Among the samples with known geographic locations, 34% originated in Asia (30 samples), while 30% and 25% of the samples originated in Europe and America, respectively. 

### 2.1. Antimicrobial Resistance Analysis 

#### 2.1.1. Phenotypic Data

The antimicrobial susceptibility testing results for our five *Ochrobactrum* isolates are presented in Table 2. We reviewed the literature for studies describing phenotypic susceptibility patterns and found minimal inhibitory concentration (MIC) data for various antimicrobials for a total of 114 isolates [17,18,19,20,22]; the MIC ranges and medians are summarized in Table 2. All five isolates were resistant to both penicillins and cephalosporins (MICs ≥ 32µg/mL). This mirrors the nearly universal resistance of these antimicrobial groups reported in the literature; all isolates reviewed were also resistant to the majority of β-lactams as well as β-lactam inhibitors, thus exhibiting an AmpC-like phenotype. Regarding carbapenems, our isolates, as well as the majority of reported isolates, showed low MIC values for this group (100% and 71%, respectively), apart from a unique cluster of carbapenem-resistant isolates in a “pseudo-bacteremia” event described in a single study [20]. Resistance to aminoglycosides appeared variable, with a median MIC value for gentamicin of 4 µg/mL (range 0.256–256) in 104 reported isolates. Of our five isolates, two exhibited low MIC values for gentamicin. With regard to other antimicrobial agents, low MIC values were reported for quinolones in both our isolates and published data (median MIC 0.25 µg/mL for ciprofloxacin), as well as for sulfonamides (median MIC 0.064 µg/mL for trimethoprim/sulfamethoxazole (TMP–SMX)).

#### 2.1.2. Resistome Analysis

##### β-Lactam Resistance

All 130 genome sequences were available for resistance gene analysis, which is summarized in Table 3. Correlating with the typical AmpC-like β-lactam resistance phenotype for this genus, 76% of the analyzed genomes possessed one variant of the *bla*_OCH__-2_ gene, the genetic determinant known to be responsible for this phenotype [22]. All of our five isolates contained one variant of the *bla*_OCH_ gene. Of note, each sample contained only one type of *bla*_OCH_ gene. Moreover, we found in 7% of the samples, another gene showing 80% homology to a reference *bla*_AmpC_ gene present in the database, originally found in *Rhodobacter sphaeroides*, which appeared to be identical in all positive samples upon multiple sequence alignment [35]. Interestingly, all sequenced samples harboring the *bla*_AmpC_-like gene were *bla*_OCH_-deficient, pointing to its potential role in β-lactam resistance. 

**Figure 1:**
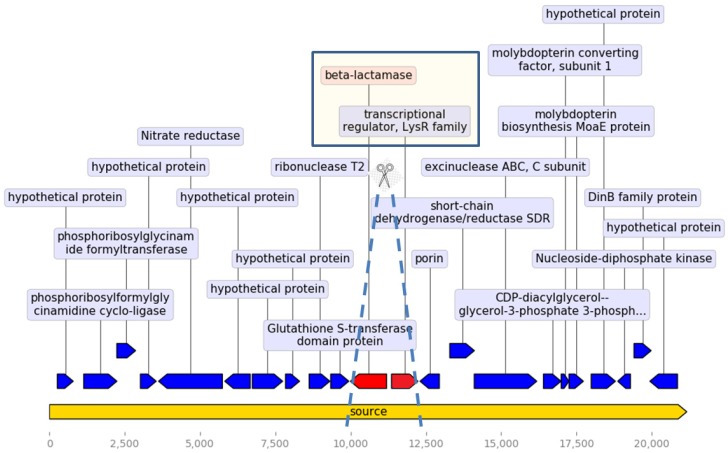
Arrangement of the genes surrounding the OCH β-lactamase and its regulator (LysR family). Dashed lines illustrate the genes that are absent in OCH-deficient samples (in red), with all other genes present (in blue).

Looking further into the differences between the samples containing *bla*_OCH_ and the *bla*_OCH_- deficient samples, we examined the genetic environment surrounding the *bla*_OCH_ gene and its divergently oriented adjacent regulatory gene of the LysR family [22]. We found 20 consecutive genes surrounding the *bla*_OCH_ gene and its regulator (placed in 10th and 11th positions), as specified in Figure 1. This set of genes was found in the vast majority of *bla*_OCH_-positive samples. Examining the *bla*_OCH_–deficient samples, we found the same genetic domain lacking only the *bla*_OCH_ gene and its regulator in 79% of such samples. No insertion sequences or repetitive elements were found within this domain.

##### Other Resistance Genes

Examining other genes associated with AMR through the Comprehensive Antibiotic Resistance Database (CARD), we found that up to 97.7% of the analyzed genomes possessed at least one gene encoding efflux pump systems or related genes, which may confer resistance to triclosan, aminoglycosides, and fluoroquinolones (Table 3). Aminoglycoside-modifying enzyme genes, namely, *aac*, *ant*, and *aph* variants, which are responsible for aminoglycoside inactivation, were found in 10.8% of the samples. Of note, none of the latter gene families were present in our five isolates, although, as mentioned above, 60% of isolates had elevated MIC values for gentamicin. Of the five new isolates, only two contained plasmids (Inc-P-like), and none contained resistance genes. 

### 2.2. Virulome Analysis 

The sequences were also analyzed for known virulence genes. Table 4 lists the genes that were present in at least 2% of the analyzed samples. Several of the genes are involved in lipid A biosynthesis, common to all Gram-negative bacteria, and found in up to 100% of the samples. Other virulence-associated genes present in the majority of the samples are involved in fatty acid biosynthesis (*fabZ*, 98%), carbohydrate metabolism (*pgm*, 97%; *cgs*, 95%), cell wall synthesis (*wbpL*, 96%), and biofilm formation (*ricA*, 95%). A gene associated with a 60 kDa chaperonin protein, *htpB*, was found in 40% of the samples. Twelve samples (9.2%) possessed *manA*, *manC*, and *wbpZ*, all of which are associated with polysaccharide synthesis. Genes associated with type IV secretion systems were found only in three of the samples analyzed. Genes for other mechanistically important virulence-associated proteins were not found in any of these samples. No virulence genes were identified within the two plasmid-related sequences found in our samples.

**Figure 2:**
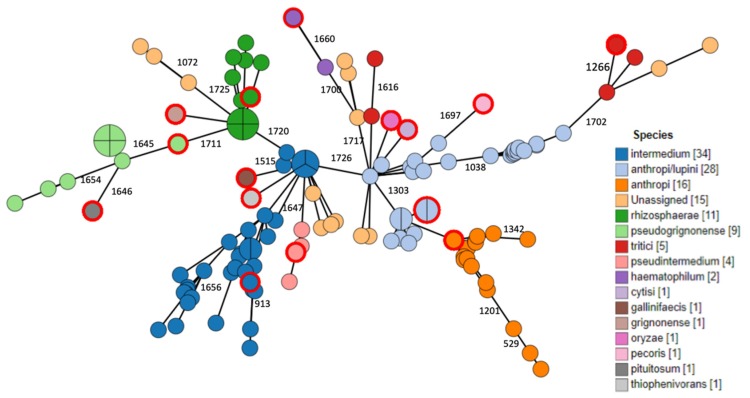
Minimum spanning tree (MST) showing ad hoc core genome multilocus sequence typing (cgMLST) analysis (1726 loci). Node color indicates species assignment using average nucleotide identity (ANI). Reference strains are denoted by an outer red ring. White nodes represent samples unassigned to any reference species.

Regarding taxonomic species identification, as there is no well-established gold standard method for species assignment within this genus, we chose to perform average nucleotide identity (ANI) on all sequences. Using this method, 88% of sequences were assigned to known species based on identity to reference strains, leaving 15 isolates unassigned (Figure 2). Due to recent changes in taxonomy [34], *O. lupini* was termed *O. lupini/anthropi*, as it was recently suggested to be a sub-species rather than a distinct unique species. Forty-four samples were classified as *O. lupini/anthropi* species, comprising 38% of all taxonomically assigned samples. A clear clustering according to species identification is evident from the tree, attesting to the reliability of the method used. Eighty-five samples had a pre-existing taxonomic assignment in their metadata, but for seven of them (8.2%), the classification based on ANI did not match the metadata. Of note, the unassigned samples formed small notable clusters, indicating that some of them may represent distinct taxonomical groups.

Another phylogenomic analysis correlated different bla_OCH_ variants with cluster types. As evident from the phylogenetic tree, two major clusters were formed by the bla_OCH_ gene type, linking bla_OCH-2_ with *O. intermedium*, and the bla_OCH_-deficient samples with *O. rhizosphaerae*.

### 2.3. Phylogenomic Analysis 

**Figure 3:**
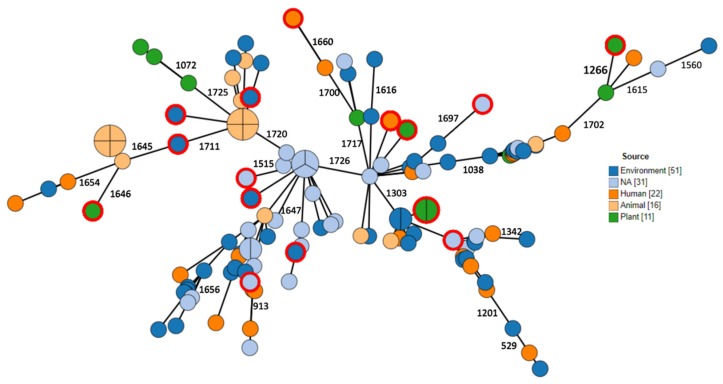
MST showing ad hoc cgMLST analysis (1726 loci). Node color indicates the source of isolation. Reference strains are denoted by an outer red ring.

**Figure 4:**
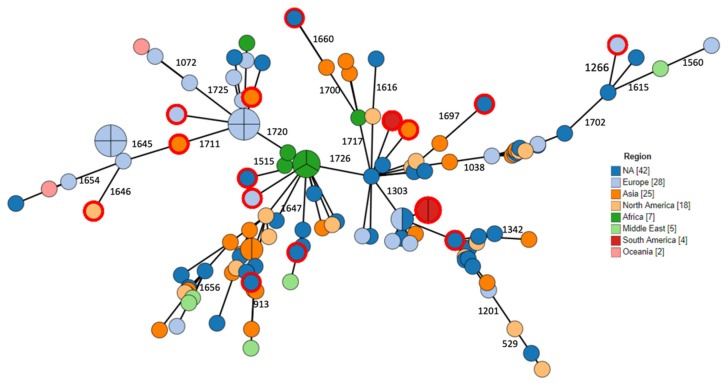
MST showing ad hoc cgMLST analysis (1726 loci). Node color indicates the geographic origin. Reference strains are denoted by an outer red ring.

Phylogenetic relations between isolates were assessed using ad hoc cgMLST consisting of 1726 loci. Data are visualized as minimum spanning tree (MST) in Figure 2, Figure 3, Figure 4. Three major clusters are apparent, further diverging into several branches. These three clusters generally correspond to the species *anthropi*, *intermedium¡,* and *rhizosphaerae*, with the *anthropi* cluster and its branches being the most prominent and diverse (Figure 2). We utilized the phylogeny analysis to explore the genetic relatedness in view of several variables. Figure 3 shows the distribution of samples with regard to their source of isolation. No apparent correlation was evident between the isolation source and any of the three clusters, with human-derived *Ochrobactrum* samples distributed all across the phylogeny. Figure 4 illustrates the geographical origin of the samples. Data were available for 67% of the isolates, which were scattered across all continents, showing no clustering of genomes according to geographical origin. 

## 3. Discussion

While *Ochrobactrum* is gaining importance as a potentially emerging pathogen, the scope of research dedicated to this genus has been limited. Few papers have attempted to extensively characterize this genus and explore its genetic background, resistome, virulome, phenotypic susceptibility, and whole-genome phylogeny. In the current meta-study, we analyzed a diverse collection of whole-genome sequences of *Ochrobactrum*, including five newly sequenced isolates. This represents the most comprehensive investigation into the genus *Ochrobactrum* to date. 

The *Ochrobactrum* genus is well known for its near-universal resistance to certain β-lactam antibiotics [18]. The genetic motif proposed as responsible for this trait is represented by the *OCH* β-lactamase genes [22]. While all isolates retrieved from our literature review and the five additional isolates were indeed resistant to penicillins and cephalosporins, we found evidence for the presence of *bla*_OCH_ genes only in 76% of the assembled genomes. Another study examining multiple *Ochrobactrum* isolates for the presence of *bla*_OCH_ genes using specific PCR, found their presence in only 83.3% of 24 *O. intermedium* and *Ochrobactrum tritici* isolates [24], while 100% exhibited an AmpC-resistance phenotype. This prompted us to search in silico for other possible β-lactamase genes in the *bla*_OCH_-deficient genomes, thus discovering a gene resembling an AmpC gene previously found in *R. sphaeroides* [35] in 7% of the samples. The fact that each sample had one variant of the *bla*_OCH_ genes, and that none of the *bla*_AmpC_-positive samples possessed another β-lactamase determinant, raises the possibility that this gene may also be responsible for the phenotypic β-lactam resistance. Unfortunately, no phenotypic data were available for the public *bla*_OCH_-deficient samples, either *bla*_AmpC_-positive or -negative, to confirm this hypothesis. Still, a notable proportion of genomes did not harbor any β-lactamase, suggesting some *Ochrobactrum* strains may lack the inherent β-lactam resistance phenotype. 

**Figure 5:**
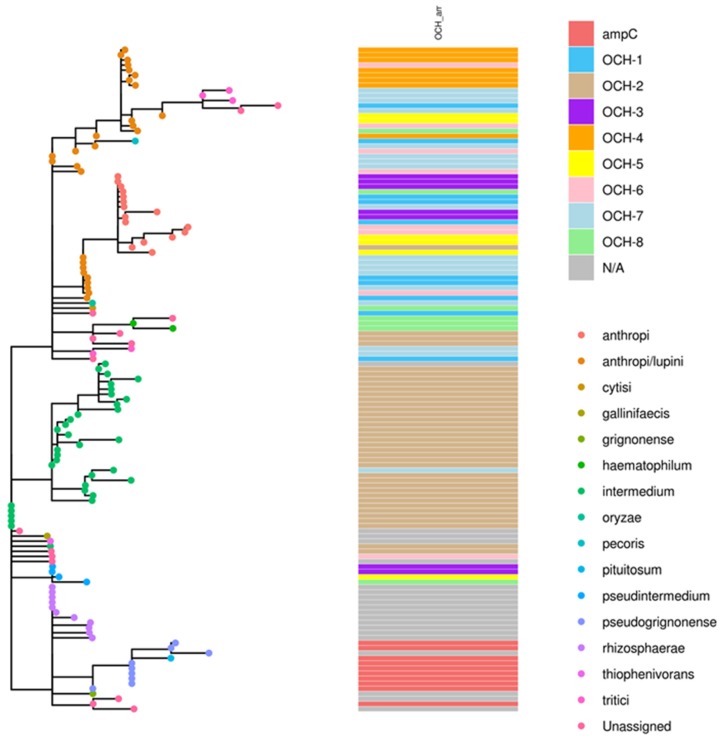
Neighbor-joining (NJ) phylogenetic tree presenting the species assignment according to ANI (node colors) and the distribution of *OCH* genes across species and phylogeny.

Analyzing the genes surrounding the *bla*_OCH_ gene and its regulator revealed a unique genetic environment, present in most *bla_OCH-_*containing genomes. Interestingly, this structure was also present without the *bla*_OCH_ gene and its companion in the *bla_OCH-_*deficient samples. The clustering of *bla*_OCH_ -deficient samples together with a distinct species, as is shown in Figure 2 and Figure 5, implies a genomic event that occurred during the evolution of this microorganism, either an acquisition or a loss of this genomic determinant, which should be further investigated. Moreover, it may allow inferring speciation based on the presence or absence of the *bla*_OCH_ gene. 

Examining AMR of other antimicrobial groups, we found that up to 97.7% of the isolates had some type of efflux pump system-related genes, but the major genetic component *triC* in this group confers resistance to a disinfectant [36], while others such as *ceoB* are rarely described as part of an apparatus conferring quinolone resistance [37]. Aminoglycoside-modifying enzymes (AME) and their corresponding genes play an important role in aminoglycoside resistance [38]. Within our group of analyzed genomes, 10.8% contained one or more AME genes. Interestingly, mining all the isolates genomes in our literature review with known AMR phenotypes, 27% showed elevated MIC values for gentamicin (>8 µg/mL). This may suggest other mechanisms of aminoglycoside resistance that may have not been described previously, but as for the discrepancy between β-lactam resistance and β-lactamase gene presence, this cannot be validated, since the assembled genome samples lacked the corresponding susceptibility data. Nevertheless, our five isolates contained no AME genes or other aminoglycosides resistance genes, although 60% showed relatively high MIC values (8 µg/mL) for gentamicin, raising questions on the genetic basis for resistance in this genus. This MIC value typically indicates intermediate resistance in other non-fermenters such as *Pseudomonas aeruginosa*, the breakpoints of which have been used by some authors to interpret antimicrobial susceptibility testing (AST) for *Ochrobactrum*. Notably, a study examining the genotype-to-phenotype correlation of a single *O. intermedium* isolate described phenotypic resistance to aminoglycosides, without underlying aminoglycoside resistance genes [39]. More phenotypic and genotypic studies are thus required in order to determine the epidemiological cut-offs for aminoglycoside resistance in this genus as well as their genetic basis.

Given the relatively low virulence content of this genus, it is not surprising that very few virulence genes were discovered in our analysis. The most common genes, found in 95%–100% of these samples likely participate in lipid A cell wall and fatty acid synthesis, carbohydrate metabolism, and biofilm formation. The chaperone protein associated with the *htpB* gene (found in 40% of samples) is considered a highly conserved and multifunctional protein [40]. In many microorganisms, this chaperonin is associated with multiple moonlighting functions, including modulation of the cytoskeleton in *Legionella* [41], cytokine synthesis in *Francisella* [42], and an insect neurotoxin in *Enterobacter* [43]. We attempted to identify genes that were more common in samples isolated from humans, thus potentially implicating proven virulence, but none of the above-mentioned genes were found to be enriched in that isolate group. Twelve of the samples we isolated contained three genes associated with polysaccharide synthesis (*manA*, *manC*, and *wbpZ)*. These 12 samples, despite having all three genes in common, represented human, animal, and environmental samples from Asia, Africa, Europe, and North America. Only 3 of the 130 samples contained a gene known to be associated with type IV secretion systems (T4SS) (*trwD*), indicating that this is an uncommon virulence mechanism for *Ochrobactrum*, as opposed to its close relative *Brucella*, in which T4SS plays a crucial role in pathogenicity [44]. 

The use of WGS allowed the construction of phylogenomic trees based on ad hoc cgMLST. This enabled an overview of the diversity and relatedness between different members of this genus. No correlation was found between the geographical origin or the source of isolation and genomic cluster types, supporting the ubiquitous environmental nature of this genus and suggesting independence of the genetic lineages with respect to specific ecological niches. The fact that no specific cluster was correlated with human origin supports the assumption that no certain species or strain are particularly host-adapted to the human niche and that the entire genus has the ability to colonize humans through incidental interaction with the environment and living animals. 

Speciation of this genus has been attempted through various molecular methods. The inability of the 16S *rRNA* gene sequence to clearly differentiate between species [29] gave rise to other techniques, including *recA*-based discrimination. Recently, a change in *Ochrobactrum* taxonomy was proposed through the combination of ANI and digital DNA–DNA hybridization (dDDH) [34]. We used FastANI on all assembled genomes in order to discriminate between different *Ochrobactrum* species. The apparent clustering according to species illustrated in Figure 2 suggests WGS-based ANI is indeed useful for species identification and assignment. Of note, samples with ANI scores of less than 95% similarity, considered unassigned to any of the known species, formed notable small clusters. Moreover, *O. tritici* appeared to cluster around two branches of *O. anthropi*. This calls for further in depth investigation, as these may represent distinct taxonomical groups. 

Our study has several limitations. Only some of the samples had accompanying metadata on the source of isolation and geography, which could introduce bias. Moreover, none of the samples had metadata on phenotypic resistance, thus no direct genotype-to-phenotype correlations could be made, apart from our five isolates. Furthermore, we assessed virulence based on sequences alone as no phenotypic assessment of virulence was available, limiting our ability to draw conclusions regarding virulence and pathogenicity potential.

## 4. Materials and Methods

### 4.1. Collection of Isolates 

Five *Ochrobactrum* isolates were recovered during prospective surveillance studies for extended-spectrum β-lactamase (ESBL)-producing *Enterobacterales* in hospitalized horses, farm horses, and zoo animals (Table 1). Fecal samples were collected using bacteriological swabs (Meus s.r.l., Piove di Sacco, Italy) and were inoculated directly into a Luria–Bertani infusion enrichment broth (Hy-Labs, Rehovot, Israel). After incubation at 37 °C (18–24 h), enriched samples were plated onto Chromagar ESBL plates (Hy-Labs, Rehovot, Israel) and incubated at 37 °C for 24 hours. Pure isolates were stored at −80 °C for further analysis. Bacterial species assignments were determined via MALDI-TOF MS analysis. AST was then assessed using VITEK 2 (bioMerieux, Mercy l’Etoile, France) using the AST cards 270 and 308. MIC values were recorded but not interpreted as susceptible or resistant, due to a lack of internationally standardized breakpoints. 

### 4.2. DNA Extraction, Library Preparation, and Sequencing 

DNA extraction was performed using the Precellys Evolution homogenizer (Bertin Technologies SAS, Montigny-le-Bretonneux, France). Each bacterial colony was homogenized using acid-washed 425–600 μm glass beads obtained from Sigma-Aldrich, St. Louis, MO, USA. The extract was then boiled for 5 minutes at 100 °C and centrifuged for 5 minutes, and the supernatant was gently transferred. DNA concentration and purity were measured using Qubit fluorometer with dsDNA high-sensitivity assay kit (Invitrogen, Waltham, Ma, USA) and NanoDrop™ One/OneC Microvolume UV–Vis Spectrophotometer (Thermo Fisher Scientific, Waltham, Ma, USA), using the ratio of absorbance at 260 nm vs. 280 nm. The genomic libraries were prepared using the Nextera Flex kit according to the manufacturer’s instructions (Illumina, San Diego, CA, USA). Sequencing was performed using iSeq 100, generating 151-bp paired-end reads (Illumina, San Diego, CA, USA). 

### 4.3. Collection of Publicly Available Genomes and Bioinformatics Analysis 

All publicly available raw reads for *Ochrobactrum* were downloaded from the Sequence Read Archive (SRA, https://www.ncbi.nlm.nih.gov/sra) database, along with the available metadata, using the reads_download component of the flowcraft pipeline (release 1.3.1, https://github.com/assemblerflow/flowcraft, with default parameters, unless stated otherwise). Additional *Ochrobactrum* assemblies were downloaded from the PATRIC database (db, https://www.patricbrc.org/; doi: 10.1093/nar/gkw1017). For the assemblies downloaded from PATRIC db, those that were reported by the PATRIC db as “POOR”-quality were removed.

Raw sequencing reads for the 5 newly sequenced and the 25 downloaded publicly available isolates underwent Quality Control (QC), assembly, and species identification using the flowcraft pipeline, including the integrity_coverage and fastqc_trimmomatic components for raw reads QC, filtering, and trimming (using FastQC and Trimmomatic) [45,46]. Read depth estimation and downsampling to 100x depth (if required) were performed with the components check_coverage and downsample_fastq (using the estimated *O. anthropi* genome size of 5 Mb and minimum coverage of 15x). Species identification for contamination and sequence quality check from the reads were performed using the kraken (version 1) component with the minikraken database (minikrakenDB2017) [47]. The filtered and trimmed reads were assembled using the spades component (SPAdes version 3.12), and the assemblies were corrected using the process_spades, assembly_mapping and pilon components. See Table S2 for sequencing data.

### 4.4. Phylogenomic Trees Construction 

The creation of an ad hoc core genome MLST scheme for *Ochrobactrum* was performed with chewBBACA [48] using the assembled *Ochrobactrum* genomes (with the Minimum BLAST score ratio (BSR) at a default 0.6, Prodigal training file trained on the *O. anthropi* ATCC 49188 reference strain complete genome [GenBank assembly accession ID: GCA_000017405.1]), producing a final cgMLST scheme consisting of 1726 loci (at 95% genome presence). The cgMLST results were visualized with appropriate metadata as a minimum spanning tree (MST), using GrapeTree (version 1.5), [49] with the MSTreeV2 method. Also using GrapeTree, a neighbour-joining (NJ) phylogenetic tree was generated from the ad hoc cgMLST profile and visualized with metadata using the R packages ggtree and ggplot2 [50].

### 4.5. Tools and Databases used for Resistance and Virulence Genes Search

For AMR and virulence genes search, we used the tool abricate (version 0.9.8) (https://github.com/tseemann/abricate), with the databases CARD (09/2019; [51]) and VFDB (09/2019; [52]). Cutoff for similarity was set at 90%. MOB-suite (version 2.1.0) (https://github.com/phac-nml/mob-suite) was used to identify and isolate plasmids for the five novel isolates assemblies (using the ‘mob_recon’ command with the ‘—run_typer’ parameter) [53]. Isolated putative plasmids were analyzed for resistance and virulence in the same manner.

### 4.6. Taxonomic Assignment Using Average Nucleotide Identity (ANI) 

Taxonomic species assignment to all newly assembled and downloaded isolates was performed with tool FastANI (https://github.com/ParBLiSS/FastANI) [54]. Briefly, each assembly was mapped to each reference strain genome (15 in total, Table S3) to find orthologous regions using the Mashmap method, for which the average nucleotide index was then calculated and used for comparison. A cutoff of 95% identity was used to assign the appropriate reference strain, where less than 95% were considered “unassigned” [34]. 

### 4.7. Literature Review for Phenotypic Resistance

We reviewed the literature to create a phenotypic profile of the *Ochrobactrum* genus. Since this genus does not have established antibiotic breakpoints, we only collected papers in which MIC values were available, and those that specified only the categorical interpretation (susceptible/resistant) were excluded from the analysis. Unfortunately, we were not able to find MIC data for any of the 125 isolates for which WGS information was available.

## 5. Conclusions

In conclusion, we herein presented a WGS-based analysis of the resistome, virulome, phylogeny, and species assignment carried out with the largest collection to date of *Ochrobactrum* genomes. Mindful that *Ochrobactrum* is increasingly being reported to cause human infection, improved understanding of its phylogeny, virulence, and resistance is becoming important. Nevertheless, our findings also suggest that *Ochrobactrum* deserves much further study and that further genomic characterization coupled with phenotypic information could shed light on its evolution, taxonomy, and antimicrobial resistance.

## Figures and Tables

**Table 1:** Features of the five new *Ochrobactrum* isolates recovered and sequenced in this study.

Isolate No.	OCH-ISR1	OCH-ISR2	OCH-ISR3	OCH-ISR4	OCH-ISR5
MALDI TOF MS identification—Best match	*O. intermedium*	*O. triciti*	*O. intermedium*	*O. intermedium*	*O. intermedium*
Origin	Horse, on admission to veterinary hospital	Horse, on admission to veterinary hospital	Healthy horse ^1^, community collection	Healthy horse ^1^, community collection	Fat sand rat (*Psammomys obesus*), Zoo collection
Comments	12-year-old gelding, gastrointestinal pathology	1-month-old filly, respiratory pathology	16-year-old healthy mare, gut colonization	9-year-old healthy mare, gut colonization	−
***WGS data:***					
Total reads	1,984,624	1,137,438	925,646	1,497,780	1,693,904
Total bases	276,000,000	160,000,000	131,000,000	203,000,000	241,000,000
% GC	57.4	55.6	57.2	57	57.3
Minimum read length	35	35	35	35	35
Average read length	139	140	141	135	142
Maximum read length	151	151	151	151	151
Mode read length	151	151	151	151	151
Average quality ^2^	35.5	35.3	35	34.9	35.6
Calculating depth	5,000,000	5,000,000	5,000,000	5,000,000	5,000,000
Sequencing depth of coverage ^3^	55x	31x	26x	40x	48x
Number of contigs	53	115	171	71	52
Total Base Pairs	3,813,327	4,758,714	4,270,779	4,807,944	4,904,698
Minimum	2.05 × 10^2^	2.00 × 10^2^	2.01 × 10^2^	200	2.03 × 10^2^
Average	86,928	41,380	249,75	67,717	94321
Maximum	912,538	500,181	476,636	874,803	830,669
N50	427,705	308,273	240,761	363,912	397,644

^1^ Farm horse; ^2^ Phred quality score; ^3^ According to 5 Mb reference.

**Table 2:** Phenotypic resistance of *Ochrobactrum* (five unpublished isolates and pooled published data). MIC, minimal inhibitory concentration.

*Antimicrobial Agent*	Measured MIC Values (µg/mL)					Reported MIC Values (µg/mL)	
	OCH-ISR1	OCH-ISR2	OCH-ISR3	OCH-ISR4	OCH-ISR5	*Median, (MIC Range)*	*Number of Isolates*
Ampicillin	≥32	≥32	≥32	≥32	≥32	256, (32–256)	101
Ampicillin–Sulbactam	≥32	≥32	≥32	≥32	≥32	32, (32–32)	5
Amoxicillin–Clavulanic Acid	≥32	≥32	≥32	≥32	≥32	256, (32–256)	101
Ticaricillin	≥128	≥128	≥64	≥128	≥128	128, (64–128)	12
Piperacillin	≥128	≥128	≥128	≥128	≥128	256, (128–256)	101
Piperacillin–tazobactam	≥128	≥128	≥128	≥128	≥1228	128, (16–256)	22
Cefalexin	≥64	≥64	≥64	≥64	≥64	64, (64–64)	5
Cefuroxime	≥64	≥64	≥64	≥64	≥64	64, (32–128)	19
Cefoxitin	≥64	≥64	≥64	≥64	≥64	96, (64–128)	12
Ceftazidime	≥64	≥64	≥64	≥64	≥64	256, (8–256)	111
Ceftriaxone	≥64	≥64	≥64	≥64	≥64	256, (4–256)	93
Ertapenem	≤0.5	≤0.5	≤0.5	≤0.5	≤0.5	0.25, (0.064–0.5)	12
Imipenem	1	0.5	0.5	1	1	2, (0.128–256)	114
Meropenem	1	0.5	0.5	1	1	0.5, (0.128–16)	21
Amikacin	≥64	4	16	16	≥64	3, (1–64)	12
Gentamicin	8	≤1	≤1	8	8	4, (0.256–256)	104
Tobramycin	≥16	≤1	≤1	≥16	≥16	16, (1–16)	5
Ciprofloxacin	≤0.25	≤0.25	0.5	≤0.25	≤0.25	0.25, (0.25–0.5)	12
Levofloxacin	0.25	0.25	0.25	0.25	0.25	0.25, (0.25–0.25)	5
Minocycline	≤1	≤1	≤1	≤1	≤1	1, (1–1)	5
Tigecycline	4	2	1	4	4	2, (0.28–4)	12
Fosfomycin	≥256	≥256	≥256	≥256	≥256	256, (256–256)	5
Nitrofurantoin	256	256	256	256	256	256, (256–256)	5
Trimethoprim/ sulfamethoxazole (TMP–SMX)	≤20	≤20	≤20	≤20	≤20	0.064, (0.064–256)	104

**Table 3:** Resistome analysis among *Ochrobactrum* genomes (n = 130). AMR, antimicrobial resistance.

Gene Name	AMR Gene Family	Drug Target	Resistance Mechanism	% of Total Sequences (n = 130)
β*-lactamase genes*				
OCH-1	OCH β-lactamase	Cephalosporin, cephamycin, monobactam	Antibiotic inactivation	7.7 (10)
OCH-2				27.7 (36)
OCH-3				4.6 (6)
OCH-4				6.2 (8)
OCH-5				4.6 (6)
OCH-6				7 (9)
OCH-7				13.1 (17)
OCH-8				5.4 (7)
Any OCH				76.2 (99)
AmpC	AmpC-type β-lactamase	Cephalosporin, penicillin	−	7 ^1^ (9)
Any β-lactamase	−	−	−	83.1 (108)
*Other resistance genes*				
*triC*	Resistance-nodulation-cell division (RND) efflux pump system and related genes	Triclosan	Antibiotic efflux complex	97.7 (127)
*ceoB*		Aminoglycosides, fluoroquinolones		96.2 (125)
*mdsB*		Phenicol antibiotics, β-lactams		42.3 (55)
*aac, ant* and *aph* variants	Aminoglycoside-modifying enzymes	Aminoglycosides	Antibiotic inactivation	10.8 ^2^ (14)

^1^ 80% homology to an existing gene in Comprehensive Antibiotic Resistance Database (CARD) database. ^2^ Percentage of samples which had at least one of the following aminoglycoside-modifying enzyme genes: *aac*(3)-IIc, *aac*(3)-IId, *aac*(6’)-Ib7, *aac*(6’)-Il, *ant*(2’’)-Ia, *ant*(3’’)-IIa, *aph*(3’’)-Ib, *aph*(3’)-Ib, *aph*(4)-Ia, *aph*(6)-Id.

**Table 4:** Virulome analysis among *Ochrobactrum* genomes (n = 130).

Gene Name	Protein	Function	% of Human Isolates (n = 22)	% of Other Isolates (n = 82)	% of Total Isolates (n = 130)
			*Percent (n)*		
*acpXL*	Acyl carrier protein	Lipid A biosynthesis	100 (22)	100 (82)	100 (130)
*htrB*	Lauroyltransferase		100 (22)	98 (80)	98 (127)
*ipx* variants	Multiple		100 ^1^ (22)	100^1^ (82)	99 ^1^ (129)
*fabZ*	Acyl carrier protein	Fatty acid biosynthesis	100 (22)	98 (80)	98 (127)
*pgm*	Phosphoglucomutase-1	Carbohydrate metabolism	95 (21)	98 (80)	97 (126)
*cgs*	Glucan synthesis		95 (21)	96 (79)	95 (123)
*wbpL*	Glucosyltransferase	Cell wall synthesis and organization	100 (22)	96 (79)	96 (125)
*ricA*	Regulator protein	Biofilm formation	100 (22)	93 (76)	95 (123)
*htpB*	Chaperone	Folding, adhesion, invasion factor	36 (8)	48 (39)	40 (52)
*man* variants	Multiple	Polysaccharide synthesis	9^2^ (2)	8.5^2^ (7)	9.2^2^ (12)
*wbpZ*	Glycosyltransferase		9 (2)	8.5 (7)	9.2 (12)
*mgtB*	Magnesium-transporting ATPase	Mediates magnesium influx	4.5 (1)	3.7 (3)	4.6 (6)
*ureB*	Urease subunit	Urea degradation	4.5 (1)	2.4 (2)	2 (3)

^1^ Percentage of samples which contained at least one of the following: *ipxA*, *ipxB*, *ipxC*, *ipxD*, or *ipxE*. ^2^ Percentage of samples which possessed *manA* and *manB* (no samples possessed only one variant).

## Supplementary Materials

The following are available online at https://www.mdpi.com/2079-6382/9/4/177/s1, Table S1: Metadata of 125 publicly available genomes, Table S2: Sequencing data for all samples; Table S3: Reference strains for ANI-based species assignment.

## Data Accessibility

Assemblies were deposited in bioproject PRJEB37004, with the following accession IDs: OCH-ISR1 (GCA_902825335); OCH-ISR2 (GCA_902825325); OCH-ISR3 (GCA_902825355); OCH-ISR4 (GCA_902825315); OCH-ISR5 (GCA_902825345).

## References

[1] Holmes B., Popoff M., Kiredjian M., Kersters K. (1988). *Ochrobactrum anthropi* gen. nov., sp. nov. from human clinical specimens and previously known as group vd. Int. J. Syst. Bacteriol..

[2] Lebuhn M., Achouak W., Schloter M., Berge O., Meier H., Barakat M., Hartmann A., Heulin T. (2000). Taxonomic characterization of *Ochrobactrum* sp. isolates from soil samples and wheat roots, and description of *Ochrobactrum tritici* sp. nov. and *Ochrobactrum grignonense* sp. nov. Int. J. Syst. Evol. Microbiol..

[3] Kampfer P., Scholz H.C., Huber B., Falsen E., Busse H.-J. (2007). *Ochrobactrum haematophilum* sp. nov. and *Ochrobactrum pseudogrignonense* sp. nov., isolated from human clinical specimens. Int. J. Syst. Evol. Microbiol..

[4] Dirksen P., Marsh S.A., Braker I., Heitland N., Wagner S., Nakad R., Mader S., Petersen C., Kowallik V., Rosenstiel P. (2016). The native microbiome of the nematode *Caenorhabditis elegans*: Gateway to a new host-microbiome model. BMC Biol..

[5] Kulkarni G., Gohil K., Misra V., Kakrani A.L., Misra S.P., Patole M., Shouche Y., Dharne M. (2017). Multilocus sequence typing of *Ochrobactrum* spp. isolated from gastric niche. J. Infect. Public Health.

[6] Cieslak T.J., Robb M.L., Drabick C.J., Fischer G.W. (1992). Catheter-associated sepsis caused by *Ochrobactrum anthropi*: Report of a case and review of related nonfermentative bacteria. Clin. Infect. Dis. Off. Publ. Infect. Dis. Soc. Am..

[7] Wheen L., Taylor S., Godfrey K. (2002). Vertebral osteomyelitis due to *Ochrobactrum anthropi*. Intern. Med. J..

[8] Ozdemir D., Soypacacı Z., Sahin I., Bicik Z., Sencan I. (2006). *Ochrobactrum anthropi* endocarditis and septic shock in a patient with no prosthetic valve or rheumatic heart disease: Case report and review of the literature. Jpn. J. Infect. Dis..

[9] Alparslan C., Yavascan O., Kose E., Sanlioglu P., Aksu N. (2013). An opportunistic pathogen in a peritoneal dialysis patient: *Ochrobactrum anthropi*. Indian J. Pediatr..

[10] Hagiya H., Ohnishi K., Maki M., Watanabe N., Murase T. (2013). Clinical characteristics of *Ochrobactrum anthropi* bacteremia. J. Clin. Microbiol..

[11] Mattos F.B., Saraiva F.P., Angotti-Neto H., Passos A.F. (2013). Outbreak of *Ochrobactrum anthropi* endophthalmitis following cataract surgery. J. Hosp. Infect..

[12] Khasawneh W., Yusef D. (2017). *Ochrobactrum anthropi* fulminant early-onset neonatal sepsis: A case report and review of literature. Pediatr. Infect. Dis. J..

[13] Gigi R., Flusser G., Kadar A., Salai M., Elias S. (2017). *Ochrobactrum anthropi*-caused osteomyelitis in the foot mimicking a bone tumor: Case report and review of the literature. J. Foot Ankle Surg..

[14] Galanakis E., Bitsori M., Samonis G., Christidou A., Georgiladakis A., Sbyrakis S., Tselentis Y. (2002). *Ochrobactrum anthropi* bacteraemia in immunocompetent children. Scand. J. Infect. Dis..

[15] Battaglia T.C. (2008). *Ochrobactrum anthropi* septic arthritis of the acromioclavicular joint in an immunocompetent 17 year old. Orthopedics.

[16] Rastogi N., Mathur P. (2017). *Ochrobactrum anthropi*: An emerging pathogen causing meningitis with sepsis in a neurotrauma patient. J. Infect. Dev. Ctries..

[17] Higgins C.S., Murtough S.M., Williamson E., Hiom S.J., Payne D.J., Russell A.D., Walsh T.R. (2001). Resistance to antibiotics and biocides among non-fermenting Gram-negative bacteria. Clin. Microbiol. Infect..

[18] Thoma B., Straube E., Scholz H.C., Al Dahouk S., Zöller L., Pfeffer M., Neubauer H., Tomaso H. (2009). Identification and antimicrobial susceptibilities of *Ochrobactrum* spp.. Int. J. Med. Microbiol..

[19] Chmelař D., Holý O., Kasáková I., Hájek M., Lazarová A., Gonzalez-Rey C., Lasák J., Raclavský V., Čižnár I. (2019). Antibiotic susceptibility and production of endotoxin by *Ochrobactrum anthropi* isolated from environment and from patients with cystic fibrosis. Folia Microbiol. (Praha).

[20] Montaña S., Fernandez J.S., Barenboim M., Hernandez M., Kayriyama C., Carulla M., Iriarte A., Ramirez M.S., Almuzara M. (2018). Whole-genome analysis and description of an outbreak due to carbapenem-resistant *Ochrobactrum anthropi* causing pseudo-bacteraemias. New Microbes New Infect..

[21] Teyssier C. (2005). Molecular and phenotypic features for identification of the opportunistic pathogens *Ochrobactrum* spp.. J. Med. Microbiol..

[22] Nadjar D., Labia R., Cerceau C., Bizet C., Philippon A., Arlet G. (2001). Molecular characterization of chromosomal class C β-Lactamase and its regulatory gene in *Ochrobactrum anthropi*. Antimicrob. Agents Chemother..

[23] Higgins C.S., Avison M.B., Jamieson L., Simm A.M., Bennett P.M., Walsh T.R. (2001). Characterization, cloning and sequence analysis of the inducible *Ochrobactrum anthropi* AmpC beta-lactamase. J. Antimicrob. Chemother..

[24] Alonso C.A., Kwabugge Y.A., Anyanwu M.U., Torres C., Chah K.F. (2017). Diversity of *Ochrobactrum* species in food animals, antibiotic resistance phenotypes and polymorphisms in the blaOCH gene. FEMS Microbiol. Lett..

[25] Poonawala H., Marrs Conner T., Peaper D.R. (2018). The brief case: Misidentification of *Brucella melitensis* as *Ochrobactrum anthropi* by Matrix-Assisted Laser Desorption Ionization-Time of Flight Mass Spectrometry (MALDI-TOF MS). J. Clin. Microbiol..

[26] Trêpa J., Mendes P., Gonçalves R., Chaves C., Brás A.M., Mesa A., Ramos I., Sá R., da Cunha J.G.S. (2018). *Brucella* vertebral osteomyelitis misidentified as an *Ochrobactrum anthropi* infection. IDCases.

[27] Horvat R.T., El Atrouni W., Hammoud K., Hawkinson D., Cowden S. (2011). Ribosomal RNA sequence analysis of *Brucella* infection misidentified as *Ochrobactrum anthropi* infection. J. Clin. Microbiol..

[28] Vila A. (2016). *Brucella suis* bacteremia misidentified as *Ochrobactrum anthropi* by the VITEK 2 system. J. Infect. Dev. Ctries..

[29] Scholz H.C., Al Dahouk S., Tomaso H., Neubauer H., Witte A., Schloter M., Kämpfer P., Falsen E., Pfeffer M., Engel M. (2008). Genetic diversity and phylogenetic relationships of bacteria belonging to the *Ochrobactrum–Brucella* group by recA and 16S rRNA gene-based comparative sequence analysis. Syst. Appl. Microbiol..

[30] Kulkarni G., Dhotre D., Dharne M., Shetty S., Chowdhury S., Misra V., Misra S., Patole M., Shouche Y. (2013). Draft genome of *Ochrobactrum intermedium* strain M86 isolated from non-ulcer dyspeptic individual from India. Gut Pathog..

[31] Liang Z., Li G., Mai B., Ma H., An T. (2019). Application of a novel gene encoding bromophenol dehalogenase from *Ochrobactrum* sp. T in TBBPA degradation. Chemosphere.

[32] Chai L., Jiang X., Zhang F., Zheng B., Shu F., Wang Z., Cui Q., Dong H., Zhang Z., Hou D. (2015). Isolation and characterization of a crude oil degrading bacteria from formation water: Comparative genomic analysis of environmental *Ochrobactrum intermedium* isolate versus clinical strains. J. Zhejiang Univ. Sci. B.

[33] Poszytek K., Karczewska-Golec J., Ciok A., Decewicz P., Dziurzynski M., Gorecki A., Jakusz G., Krucon T., Lomza P., Romaniuk K. (2018). Genome-guided characterization of *Ochrobactrum* sp. POC9 enhancing sewage sludge utilization—Biotechnological potential and biosafety considerations. Int. J. Environ. Res. Public. Health.

[34] Gazolla Volpiano C., Hayashi Sant’Anna F., Ambrosini A., Brito Lisboa B., Kayser Vargas L., Passaglia L.M.P. (2019). Reclassification of *Ochrobactrum lupini* as a later heterotypic synonym of *Ochrobactrum anthropi* based on whole-genome sequence analysis. Int. J. Syst. Evol. Microbiol..

[35] Baumann M., Simon H., Schneider K.H., Danneel H.J., Küster U., Giffhorn F. (1989). Susceptibility of *Rhodobacter sphaeroides* to beta-lactam antibiotics: Isolation and characterization of a periplasmic beta-lactamase (cephalosporinase). J. Bacteriol..

[36] Ntreh A.T., Weeks J.W., Nickels L.M., Zgurskaya H.I. (2016). Opening the channel: The two functional interfaces of *Pseudomonas aeruginosa* opmH with the triclosan efflux pump triABC. J. Bacteriol..

[37] Guglierame P., Pasca M.R., De Rossi E., Buroni S., Arrigo P., Manina G., Riccardi G. (2006). Efflux pump genes of the resistance-nodulation-division family in *Burkholderia cenocepacia* genome. BMC Microbiol..

[38] Shaw K.J., Rather P.N., Hare R.S., Miller G.H. (1993). Molecular genetics of aminoglycoside resistance genes and familial relationships of the aminoglycoside-modifying enzymes. Microbiol. Rev..

[39] Johnning A., Moore E.R.B., Svensson-Stadler L., Shouche Y.S., Larsson D.G.J., Kristiansson E. (2013). The acquired genetic mechanisms of a multi-resistant bacterium isolated from a treatment plant receiving wastewater from antibiotic production. Appl. Environ. Microbiol..

[40] Henderson B., Fares M.A., Lund P.A. (2013). Chaperonin 60: A paradoxical, evolutionarily conserved protein family with multiple moonlighting functions. Biol. Rev. Camb. Philos. Soc..

[41] Chong A., Lima C.A., Allan D.S., Nasrallah G.K., Garduño R.A. (2009). The purified and recombinant *Legionella pneumophila* chaperonin alters mitochondrial trafficking and microfilament organization. Infect. Immun..

[42] Noah C.E., Malik M., Bublitz D.C., Camenares D., Sellati T.J., Benach J.L., Furie M.B. (2010). GroEL and lipopolysaccharide from *Francisella tularensis* live vaccine strain synergistically activate human macrophages. Infect. Immun..

[43] Yoshida N., Oeda K., Watanabe E., Mikami T., Fukita Y., Nishimura K., Komai K., Matsuda K. (2001). Protein function. Chaperonin turned insect toxin. Nature.

[44] Ke Y., Wang Y., Li W., Chen Z. (2015). Type IV secretion system of *Brucella* spp. and its effectors. Front. Cell. Infect. Microbiol..

[45] Andrews S. Babraham Bioinformatics—FastQC A Quality Control tool for High Throughput Sequence Data. http://www.bioinformatics.babraham.ac.uk/projects/fastqc/.

[46] Bolger A.M., Lohse M., Usadel B. (2014). Trimmomatic: A flexible trimmer for Illumina sequence data. Bioinforma. Oxf. Engl..

[47] Wood D.E., Salzberg S.L. (2014). Kraken: Ultrafast metagenomic sequence classification using exact alignments. Genome Biol..

[48] Silva M., Machado M.P., Silva D.N., Rossi M., Moran-Gilad J., Santos S., Ramirez M., Carriço J.A. (2018). chewBBACA: A complete suite for gene-by-gene schema creation and strain identification. Microb. Genomics.

[49] Zhou Z., Alikhan N.-F., Sergeant M.J., Luhmann N., Vaz C., Francisco A.P., Carriço J.A., Achtman M. (2018). GrapeTree: Visualization of core genomic relationships among 100,000 bacterial pathogens. Genome Res..

[50] Wickham H. (2016). Ggplot2: Elegant Graphics for Data Analysis.

[51] Jia B., Raphenya A.R., Alcock B., Waglechner N., Guo P., Tsang K.K., Lago B.A., Dave B.M., Pereira S., Sharma A.N. (2017). CARD 2017: Expansion and model-centric curation of the comprehensive antibiotic resistance database. Nucleic Acids Res..

[52] Chen L., Zheng D., Liu B., Yang J., Jin Q. (2016). VFDB 2016: Hierarchical and refined dataset for big data analysis—10 years on. Nucleic Acids Res..

[53] Robertson J., Nash J.H.E. (2018). MOB-suite: Software tools for clustering, reconstruction and typing of plasmids from draft assemblies. Microb. Genomics.

[54] Jain C., Rodriguez-R L.M., Phillippy A.M., Konstantinidis K.T., Aluru S. (2018). High throughput ANI analysis of 90K prokaryotic genomes reveals clear species boundaries. Nat. Commun..

