# Supplementary material for: Genomic Characterization of Antimicrobial Resistance, Virulence, and Phylogeny of the Genus Ochrobactrum
Source: Antibiotics (Basel). 2020 Apr 13;9(4):177. doi: 10.3390/antibiotics9040177 (PMC7235858; doi:10.3390/antibiotics9040177)
Supplement: Supplementary file 1 [file antibiotics-09-00177-s001.pdf]

Table S1. *Metadata of 125 publicly available genomes*

Genome ID	Species using ANI	Source of isolation	Isolation Country	Region
1151114.3	anthropi/lupini	NA	NA	NA
1234597.14 / SRR2961886	intermedium	Human	India	Asia
1234597.4	intermedium	Human	India	Asia
1337887.3	intermedium	Human	India	Asia
1390361.3	intermedium	Environment	India	Asia
1437448.3	rhizosphaerae	Environment	China	Asia
1444311.3	intermedium	Environment	China	Asia
1449061.3	anthropi/lupini	NA		NA
1707152.3	intermedium	Environment	India	Asia
1720558.3	anthropi/lupini	Environment	China	Asia
1827297.5 / SRR6003490	Unassigned	Animal	Germany	Europe
1830124.3	Unassigned	Environment	China	Asia
1848699.3 / SRR6003504	rhizosphaerae	Animal	Germany	Europe
1848700.3 / SRR6003493	rhizosphaerae	Animal	Germany	Europe
1848701.3 / SRR6003492	rhizosphaerae	Animal	Germany	Europe
1848702.3 / SRR6003494	rhizosphaerae	Animal	Germany	Europe
1848703.3 / SRR6003486	rhizosphaerae	Animal	Germany	Europe
1848704.3 / SRR6003453	anthropi/lupini	Animal	Germany	Europe
1920636.3	Unassigned	Environment	Argentina	South America
1947010.3	intermedium	Environment	United States	North America
1947011.3	intermedium	Environment	United States	North America
2033031.3 / SRR6003525	pseudogrignonense	Animal	Germany	Europe
2038104.3	intermedium	NA	Nigeria	Africa
2038105.3	intermedium	NA	Nigeria	Africa
2038106.3	intermedium	NA	Nigeria	Africa
2038107.3	intermedium	NA	Nigeria	Africa
2038108.3	intermedium	NA	Nigeria	Africa
2038109.3	intermedium	NA	Pakistan	Asia
2038110.3	intermedium	NA	Pakistan	Asia
2038111.3	intermedium	NA	Pakistan	Asia
2042474.3 / SRR6003496	rhizosphaerae	Animal	Germany	Europe
215590.7	gallinifaecis	NA	NA	NA
**Genome ID**	**Species using ANI**	**Source of isolation**	**Isolation Country**	**Region**
2203419.3	Unassigned	Environment	Poland	Europe
2316647.3 / SRR7867373	anthropi/lupini	Environment	China	Asia
2448455.3	Unassigned	Environment	South Korea	Asia

2479765.3	Unassigned	NA	China	Asia
2485134.3 / SRR8392523	Unassigned	NA	USA	North America
255457.3	anthropi/lupini	Plant	Argentina	South America
255457.6	anthropi/lupini	Plant	Argentina	South America
2583453.3	Unassigned	Environment	NA	NA
2650567.3	intermedium	Human	NA	NA
2650568.3	haematophilum	Human	Afghanistan	Asia
271865.3	Unassigned	Plant	Netherlands	Europe
271865.6	Unassigned	Plant	NA	Europe
335286.3	oryzae	Human	Brazil	South America
360313.3	pseudogrignonense	Environment	Australia	Oceania
370111.3	pseudintermedium	Environment	NA	NA
407152.3 / SRR5003446	cytisi	Plant	Russia	Asia
419474.10	haematophilum	Human	NA	NA
419474.7	Unassigned	Plant	Nigeria	Africa
419474.9	Unassigned	NA	NA	NA
419475.13	pseudogrignonense	Human	Sweden	Europe
419475.15 / SRR6003466	pseudogrignonense	Animal	Germany	Europe
419475.16 / SRR6003449	pseudogrignonense	Animal	Germany	Europe
419475.17 / SRR6003478	pseudogrignonense	Animal	Germany	Europe
419475.19	pseudogrignonense	Human	NA	NA
419475.3	pseudogrignonense	Environment	Malaysia	Asia
42190.13 / SRR6485881	intermedium	Environment	NA	NA
439375.7	anthropi	NA	NA	NA
529.12	tritici	Environment	United States	North America
529.33	anthropi/lupini	Environment	United States	North America
529.35	anthropi	Environment	United States	North America
529.36	anthropi/lupini	Environment	United States	North America
529.37	anthropi/lupini	Environment	United States	North America
529.38	anthropi	Environment	United States	North America
**Genome ID**	**Species using ANI**	**Source of isolation**	**Isolation Country**	**Region**
529.39	anthropi	Environment	United States	North America
529.40	anthropi/lupini	Environment	United States	North America
529.41	anthropi	Environment	United States	North America
529.42	anthropi/lupini	Environment	United States	North America
529.6	anthropi/lupini	Environment	China	Asia
529.62	anthropi/lupini	Environment	Spain	Europe
529.63 / SRR6487964	anthropi	Environment	NA	NA
529.64	anthropi	NA	NA	NA

529.7	anthropi	NA	NA	NA
529.70	anthropi	Environment	Pakistan	Asia
529.71	anthropi	Environment	Pakistan	Asia
529.78	anthropi/lupini	Environment	NA	NA
529.79	anthropi	Environment	NA	NA
529.8	anthropi/lupini	Environment	Vietnam	Asia
529.80	anthropi/lupini	Human	NA	NA
529.81	anthropi	Human	NA	NA
529.82	anthropi/lupini	Human	NA	NA
529.83	anthropi	Human	NA	NA
529.84	anthropi/lupini	Human	NA	NA
529.85	anthropi	Human	NA	NA
529.86	anthropi	Human	USA	North America
529.87	anthropi/lupini	Environment	Sweden	Europe
529.88	anthropi/lupini	Environment	NA	NA
529.89	anthropi/lupini	Human	Norway	Europe
529.90	anthropi/lupini	Human	NA	NA
529.91	anthropi	Human	Sweden	Europe
529.92	anthropi/lupini	NA	NA	NA
529.93	anthropi/lupini	Animal	Czech Republic	Europe
529.94	anthropi/lupini	Environment	France	Europe
529.95	anthropi/lupini	Plant	South Korea	Asia
571254.3	Unassigned	Plant	Australia	Oceania
571255.10 / SRR6003475	rhizosphaerae	Animal	Germany	Europe
571255.8	thiophenivorans	Environment	Germany	Europe

**Genome ID**	**Species using ANI**	**Source of isolation**	**Isolation Country**	**Region**
571256.17	rhizosphaerae	Environment	Tunisia	Africa
571256.25	rhizosphaerae	Environment	NA	NA
571256.9	pituitosum	Plant	United States	North America
586218.3 / SRR8450062	rhizosphaerae	Environment	NA	NA
641118.3	intermedium	NA	NA	NA
867683.3	pecoris	NA	NA	NA
936132.4 / SRR3929697	pseudintermedium	NA	NA	NA
94625.13	intermedium	NA	NA	NA
94625.19	intermedium	Environment	NA	NA
94625.20	intermedium	Environment	France	Europe
94625.21	intermedium	Environment	NA	NA
94625.22	intermedium	Human	NA	NA
94625.23	intermedium	Human	NA	NA

94625.24	intermedium	NA	NA	NA
94625.25	intermedium	Environment	USA	North America
94625.5	intermedium	Environment	China	Asia
94625.6	intermedium	Animal	United States	North America
94625.7	intermedium	Environment	Saudi Arabia	Asia
94625.9	intermedium	Environment	United States	North America
94626.10	tritici	Plant	NA	NA
94626.7	tritici	Human	NA	NA
94626.8	tritici	Environment	NA	NA
94626.9	tritici	Plant	France	Europe
94627.10	grignonense	Environment	France	Europe
SRR1798201	anthropi/lupini	NA	NA	NA
SRR3928092	pseudintermedium	NA	NA	NA
SRR7168035	Unassigned	NA	NA	NA

Table S2. *Sequencing data for all samples*

Name	No.	Base Pairs	OK	NS	Gaps	Min	Avg	Max	N50
OCH-ISR1	53	4607187	4607187	0	0	205	86928	912538	427705
OCH-ISR2	115	4758714	4758714	0	0	200	41380	500181	308273
OCH-ISR3	171	4270779	4270779	0	0	201	24975	476636	240761
OCH-ISR4	71	4807944	4807944	0	0	200	67717	874803	363912
OCH-ISR5	52	4904698	4904698	0	0	203	94321	830669	397644
1151114.3	47	4712978	4712978	0	0	205	100276	726895	316618
1234597.4	148	5188688	5188688	0	0	392	35058	224875	78024
1337887.3	378	4808223	4808223	0	0	202	12720	71323	19057
1390361.3	54	4839722	4839722	0	0	1008	89624	509970	261374
1437448.3	198	5245769	5245769	0	0	547	26493	471969	107455
1444311.3	95	4800175	4800175	0	0	501	50528	671480	194604
1449061.3	16	4624630	4624630	0	0	2205	289039	1577432	1053978
1707152.3	11	4660061	4658888	1173	0	733	423641	2550118	2550118
1720558.3	24	4630364	4627175	3189	0	1071	192931	1135771	585052
1830124.3	36	4951322	4951322	0	0	595	137536	1062924	467496
1920636.3	65	5253132	5253132	0	0	1037	80817	445701	242240
1947010.3	111	4571323	4567906	3417	0	2565	41183	431507	88603
1947011.3	23	4544042	4542736	1306	0	6110	197567	755421	430760
2038104.3	13	4935855	4935855	0	0	22541	379681	1951974	855839
2038105.3	19	4937528	4937528	0	0	18479	259869	1698597	946216
2038106.3	18	4936674	4936674	0	0	20660	274259	1419977	451323
2038107.3	18	4936858	4936858	0	0	2989	274269	1330586	495115
2038108.3	18	4938583	4938583	0	0	1704	274365	1614757	999630
2038109.3	22	4855883	4855883	0	0	708	220721	1307228	793849
2038110.3	23	4856529	4856529	0	0	1667	211153	1037751	671229
2038111.3	24	4860377	4860377	0	0	782	202515	1124918	431306
215590.7	37	3742416	3742406	10	0	1225	101146	495126	373399
2203419.3	255	4969575	4969575	0	0	204	19488	507722	182332
2448455.3	48	5024120	5024120	0	0	1516	104669	888782	359264
2479765.3	43	4760804	4760804	0	0	1107	110716	909449	262310
255457.3	65	5582813	5582811	2	0	330	85889	624561	209686
255457.6	100	5430960	5430960	0	0	217	54309	624829	208867
**Name**	**No.**	**Base Pairs**	**OK**	**NS**	**Gaps**	**Min**	**Avg**	**Max**	**N50**
2583453.3	51	4602745	4601991	754	0	293	90249	1011582	484801
2650567.3	15	4611544	4611544	0	0	233	307436	1585708	1454747
2650568.3	56	4695774	4694721	1053	0	536	83853	689592	332982
271865.3	4	5645291	5645291	0	0	19701	1411322	2585393	2008185
271865.6	77	5052642	5052642	0	0	600	65618	445155	173928
335286.3	289	4467006	4467006	0	0	201	15456	328852	137902

360313.3	146	4971228	4971215	13	0	530	34049	350235	51173
370111.3	45	4394636	4394636	0	0	253	97658	531236	231484
419474.1	55	5503262	5503262	0	0	1005	100059	597669	276154
419474.7	5	5482303	5482303	0	0	181071	1096460	2602474	1396538
419474.9	3	4910164	4910164	0	0	878348	1636721	2596063	2596063
419475.13	53	5531562	5531557	5	0	307	104369	803971	250510
419475.19	35	4979464	4979464	0	0	219	142270	2159127	516269
419475.3	2	4992805	4992805	0	0	1042249	2496402	3950556	3950556
529.12	17	4538068	4538068	0	0	2482	266945	954155	457312
529.33	35	4977838	4976691	1147	0	11382	142223	691589	241376
529.35	50	4910867	4910245	622	0	3412	98217	645142	172659
529.36	294	4124508	4065967	58541	0	2600	14028	98123	21770
529.37	33	4802997	4802648	349	0	3415	145545	598764	313066
529.38	331	3813327	3767972	45355	0	2502	11520	237540	18108
529.39	28	4599886	4599395	491	0	4427	164281	699371	250386
529.4	36	5074424	5073489	935	0	5391	140956	813451	346568
529.41	64	4796060	4794106	1954	0	3246	74938	592744	154656
529.42	26	4584499	4583647	852	0	6512	176326	588006	313749
529.6	43	5277818	5277767	51	0	744	122739	1032997	475065
529.62	26	4904693	4904693	0	0	241	188642	1441125	688210
529.64	5	5239764	5239764	0	0	18066	1047952	2887299	2887299
529.7	4	4901165	4901165	0	0	106739	1225291	2708454	2708454
529.7	41	5066712	5066604	108	0	500	123578	1080236	458212
529.71	79	5020564	5020267	297	0	536	63551	1063111	246939
529.78	12	4806735	4806735	0	0	1223	400561	1323744	735006
529.79	70	4978335	4978335	0	0	214	71119	974321	372678
529.8	74	4904177	4903623	554	0	334	66272	353539	169533

**Name**	**No.**	**Base Pairs**	**OK**	**NS**	**Gaps**	**Min**	**Avg**	**Max**	**N50**
529.8	10	4808150	4808150	0	0	1225	480815	1332311	1072008
529.81	20	4854606	4854606	0	0	853	242730	1080266	820730
529.82	23	5007797	5007797	0	0	244	217730	1823879	1110195
529.83	76	5191372	5191372	0	0	248	68307	1079029	827187
529.84	161	5313857	5313857	0	0	218	33005	447321	175282
529.85	41	4912846	4912846	0	0	655	119825	1010885	372678
529.86	33	4958302	4958302	0	0	236	150251	1179809	1000334
529.87	37	4904887	4904887	0	0	216	132564	1137031	734175
529.88	31	4797768	4797768	0	0	654	154766	988204	450509
529.89	51	5446543	5446543	0	0	206	106794	1061732	557659
529.9	27	5142012	5142012	0	0	8438	190444	893633	380842
529.91	65	5179000	5179000	0	0	236	79676	1051120	489086
529.92	72	5119314	5119314	0	0	247	71101	624101	215355
529.93	21	4839150	4839150	0	0	246	230435	1289013	810703

529.94	17	4813838	4813838	0	0	221	283166	1323418	735006
529.95	2	4736879	4736879	0	0	2090924	2368439	2645955	2645955
571254.3	36	4904011	4904011	0	0	314	136222	978742	440158
571255.8	77	4364783	4364781	2	0	362	56685	679617	168659
571256.17	9	4885407	4884207	1200	0	2471	542823	2480539	2480539
571256.25	60	5170436	5170436	0	0	429	86173	908092	382153
571256.9	4	5467370	5467370	0	0	332144	1366842	2385017	1716190
641118.3	4	4725392	4725392	0	0	60172	1181348	2604449	2604449
867683.3	61	5057340	5057340	0	0	1057	82907	683678	283806
94625.13	3	4727886	4727886	0	0	59782	1575962	2608764	2608764
94625.19	67	4444120	4444120	0	0	215	66330	1020404	681751
94625.2	60	4851039	4851039	0	0	205	80850	988702	495364
94625.21	36	4531554	4531554	0	0	212	125876	1109134	897116
94625.22	99	4644694	4644694	0	0	200	46916	1131370	425471
94625.23	26	4479370	4479370	0	0	270	172283	987372	498616
94625.24	83	4846268	4846268	0	0	207	58388	864912	470435
94625.25	33	4975626	4975626	0	0	619	150776	1160471	513832
94625.5	48	3938898	3938898	0	0	236	82060	982890	419558
94625.6	47	4703586	4703586	0	0	240	100076	907708	669681
**Name**	**No.**	**Base Pairs**	**OK**	**NS**	**Gaps**	**Min**	**Avg**	**Max**	**N50**
94625.7	38	4913201	4913201	0	0	1164	129294	636235	419572
94625.9	10	4076837	4075896	941	0	130074	407683	769395	579011
94626.1	74	5887706	5887706	0	0	6915	79563	497894	135011
94626.7	141	4968050	4968050	0	0	200	35234	329778	108744
94626.8	44	5025724	5025724	0	0	207	114221	1096912	955119
94626.9	153	5203002	5203002	0	0	200	34006	444523	142456
94627.1	169	4838271	4838254	17	0	311	28628	842127	259681
SRR1798201	13	4621112	4621112	0	0	934	355470	2185865	816039
SRR2961886	121	4681757	4681748	9	0	212	38692	455207	96999
SRR3928092	82	4219778	4219778	0	0	208	51460	360585	209220
SRR7168035	35	5480168	5480168	0	0	252	156576	856464	590688
SRR6003490	3	5399770	5399770	0	0	269404	1799923	2834956	2834956
SRR6003504	20	4632136	4632136	0	0	1330	231606	1026751	942115
SRR6003492	3	4657557	4657557	0	0	610475	1552519	2399914	2399914
SRR6003492	23	4632353	4632353	0	0	1344	201406	942048	715679
SRR6003494	21	4631732	4631666	66	0	1128	220558	1026536	929109
SRR6003478	106	4820303	4820303	0	0	1031	45474	890616	302370
SRR6003453	3	4825002	4825002	0	0	141660	1608334	2658027	2658027
SRR6129961	15	4941717	4941217	500	0	1431	329447	1136207	537919
SRR6003496	41	5058358	5058358	0	0	1009	123374	940562	760690
SRR6487964	15	4564289	4564268	21	0	319	304285	754092	624002
SRR8392523	47	5052428	5052324	104	0	1241	107498	1095066	592911

SRR5003446	192	5965364	5964401	963	0	249	31069	453184	140216
SRR6003466	3	4974493	4974493	0	0	826252	1658164	2438388	1709853
SRR6003449	17	4936467	4935982	485	0	1431	290380	1136163	537756
SRR6003478	19	4939200	4938898	302	0	1022	259957	1136561	402427
SRR6485881	576	3440061	3406226	33835	0	2509	5972	23925	6640
SRR6487964	646	3929638	3881146	48492	0	2508	6083	27333	6876
SRR6003475	3	4657555	4657555	0	0	610473	1552518	2399914	2399914
SRR8450062	48	4365194	4365194	0	0	1223	90941	1387448	493634
SRR3929697	60	4215398	4214849	549	0	1103	70256	382041	214995

Table S3. *Reference strains for ANI based species assignment*

Reference strain	Genome ID
Ochrobactrum pituitosum strain AA2	571256.9
Ochrobactrum rhizosphaerae strain SJY1	1437448.3
Ochrobactrum grignonense strain OgA9a	94627.10
Ochrobactrum cytisi strain IPA7.2	407152.3
Ochrobactrum pseudogrignonense strain K8	419475.3
Ochrobactrum thiophenivorans strain DSM 7216	571255.8
Ochrobactrum oryzae strain OA447	335286.3
Ochrobactrum tritici strain LMG 18957	94626.9
Ochrobactrum anthropi ATCC 49188	439375.7
Ochrobactrum pecoris strain 08RB2639	867683.3
Ochrobactrum lupini strain LUP21	255457.3
Ochrobactrum pseudintermedium strain CCUG 34735	370111.3
Ochrobactrum gallinifaecis strain ISO 196	215590.7
Ochrobactrum intermedium LMG 3301	641118.3
Ochrobactrum haematophilum strain CCUG 38531	419474.10
